# Recent Advances Toward Transparent Methane Emissions Monitoring: A Review

**DOI:** 10.1021/acs.est.2c02136

**Published:** 2022-11-23

**Authors:** Broghan M. Erland, Andrew K. Thorpe, John A. Gamon

**Affiliations:** † Department of Earth and Atmospheric Sciences, University of Alberta, Edmonton, T6G 2R3, Canada; ‡ School of Natural and Environmental Sciences, Newcastle University, Newcastle Upon Tyne NE1 7RU, U.K.; § Jet Propulsion Laboratory, California Institute of Technology, Pasadena, California 91109, United States; ∥ School of Natural Resources, University of Nebraska-Lincoln, Lincoln, Nebraska 68583, United States

**Keywords:** methane emissions, monitoring technology, spectroscopy, top-down, bottom-up, remote sensing

## Abstract

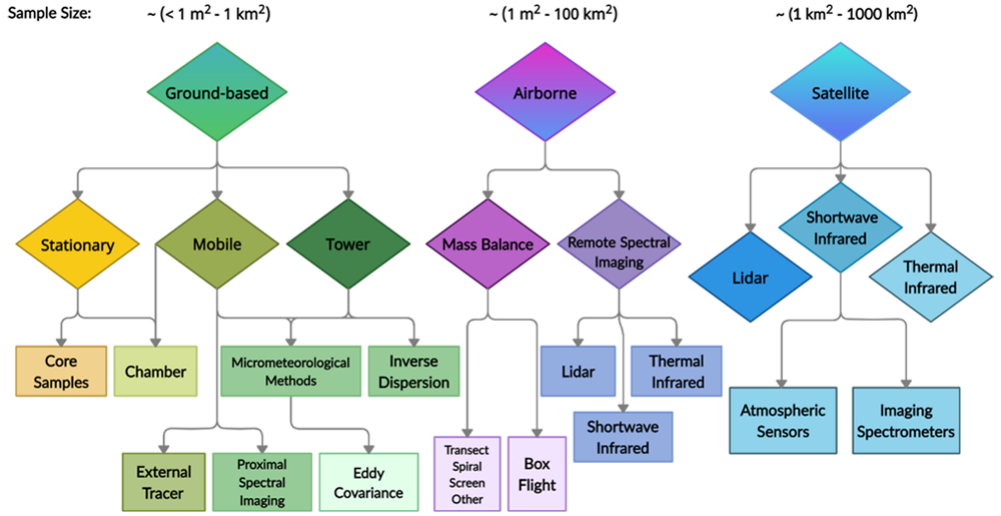

Given that anthropogenic greenhouse gas (GHG) emissions must be immediately reduced to avoid drastic increases in global temperature, methane emissions have been placed center stage in the fight against climate change. Methane has a significantly larger warming potential than carbon dioxide. A large percentage of methane emissions are in the form of industry emissions, some of which can now be readily identified and mitigated. This review considers recent advances in methane detection that allow accurate and transparent monitoring, which are needed for reducing uncertainty in source attribution and evaluating progress in emissions reductions. A particular focus is on complementary methods operating at different scales with applications for the oil and gas industry, allowing rapid detection of large point sources and addressing inconsistencies of emissions inventories. Emerging airborne and satellite imaging spectrometers are advancing our understanding and offer new top-down assessment methods to complement bottom-up methods. Successfully merging estimates across scales is vital for increased certainty regarding greenhouse gas emissions and can inform regulatory decisions. The development of comprehensive, transparent, and spatially resolved top-down and bottom-up inventories will be crucial for holding nations accountable for their climate commitments.

## 1 Methane Abatement from Large Emitters Is Essential to Combatting Global Warming

The rate of warming of the Earth is greater now than at any other period over the last 22,000 years, and the scientific consensus is that this warming is an unequivocal consequence of human activity since the Industrial Revolution.^1−10^ The continued growth of greenhouse gas (GHG) emissions must be curbed to avoid drastic increases in global temperature (>8.5 °C) by the end of this century, and emissions must be immediately reduced to have a 66% chance of keeping global warming below 2 °C.^11,12^ After carbon dioxide, methane is the second greatest contributor to anthropogenic climate warming and has 20–80 times the warming potential of carbon dioxide depending on the time frame considered.^7,8,13^ Global methane concentrations have more than doubled from an approximate average of 695 ppb between 1000 to 1800 to an estimate of 1866 ppb in 2018.^1,8,9^ Anthropogenic methane emissions have been dramatically growing over the past decade.^10,14^ Decreases in methane emissions are a highly efficient way to immediately combat global warming.^3,7,15,16^

There has been a variable growth rate of anthropogenic methane emissions over the last few decades with an unclear cause.^14,17^ According to isotopic signature measurements of ice core and accumulated snow samples to assess preindustrial methane levels, the extent of the increase in anthropogenic fossil fuel methane emissions may be underestimated by 25–40%.^10,18^ Even if net-zero emissions were attained, atmospheric methane concentrations are estimated to continue to increase for three decades before stabilizing due to chemical feedbacks and the potential declining oxidative capacity of the global atmosphere.^17,19^ Global warming itself is increasing methane emissions from natural sources,^3,5,8,10,19^ and these may represent difficult targets for reduction. Some anthropogenic methane emissions, such as those from the oil and gas sector, represent relatively tractable paths for significant and rapid GHG reduction. Recent advances in the technology for monitoring methane emissions now offer a clear path for significant, rapid, and verifiable GHG monitoring as part of a comprehensive strategy for GHG reductions. With these recent advances, sources of large abatable methane emissions, such as those found from pipeline leaks or large industrial facilities, can now be targeted to immediately combat climate change.^20,21^

National inventories are essential for creating a global picture of climate change, evaluating the effectiveness of climate change policies, and generating international pressure for action. International climate accords such as Kyoto (1997) and Paris (2005) have seen nations enthusiastically sign and then abandon pledges as governments’ commitments shift. Despite 30 years of collective agreements, carbon dioxide emissions have continued to rise at increasing rates, illustrating the failure of current initiatives and the importance of methane as a logical target for quick action.^22^ Nations that signed the United Nations Framework Convention on Climate Change (UNFCCC) agreed to use comparable methodologies to compile national inventories of anthropogenic emissions.

Accurate inventories and effective emissions reductions will require accurate and extensive monitoring, both to locate large emissions sources, and to verify that emissions reductions are effective. Bottom-up and top-down approaches to measuring methane fluxes can have varying definitions depending on the spatial scale and context of estimation,^23^ and both are essential parts of an effective monitoring strategy. In general, bottom-up methods can be described as measurements to obtain component or site-specific emission data that are then extrapolated by singular factor to estimate regional emissions.^24,25^ Top-down measurements attempt to constrain the overall budget of atmospheric GHG concentrations by sampling at larger spatial scales and utilizing modeling tools to infer point and area source emissions estimates.^23,25^ Independent estimates of fluxes used to derive the sources and sinks of the global methane budgets do not always agree with each other.^8^ Some of the largest atmospheric methane budget uncertainties arise from the differences in anthropogenic bottom-up inventory estimates and top-down budget estimates.^8,17^

Independent measurements provide a vital check on reported values as industry inventory estimates have often been noted as underestimating emissions.^26,27,36,37,28−35^ In a study systematically comparing 20 years of top-down and bottom-up estimates of anthropogenic methane emissions from the U.S. natural gas and oil sectors, official bottom-up derived inventories were found to consistently under-report methane emissions.^26^ Many factors contribute to this underestimation. The distribution of regional emissions is often mischaracterized so that the extent of emissions from “super-emitters” is under-represented and potentially biased. Large confidence intervals of estimates can further contribute to under-reporting.^26^ Inventories can also be incomplete and contradictory due to methodological issues. For example, the exclusion of abandoned wells, unknown sources, and emitters below detection limits as well as a lack of modern sampling technologies can all contribute to an under-estimation of methane emissions.^26,29,38^ Currently some heavy oil production sites report zero methane emissions despite measurable sporadic releases still occurring, as reporting is only necessary when emissions are greater than a given threshold.^29^ Upscaling using bottom-up methods that do not account for super-emitters, large sporadic emissions, and leaks are bound to produce inventories that underestimate emissions.^37,40^ Until recently, due to technological restrictions and limited access to facilities, industry inventories have been relied upon to provide essential summarized information used as a foundation for regulation.^39^ Increased transparency using remote sensing methods will improve certainty and address under-estimation of emissions inventories while holding industry more accountable to their emissions.

This review provides an overview of the wide range of current top-down and bottom-up methods for the development and continual refinement of a transparent methane monitoring systems, with a particular focus on emerging remote sensing and sampling at multiple spatiotemporal scales. Characterizing the strengths and weaknesses of ground-based, airborne, and satellite methods and the potential for synergies between them is an essential step toward creating exhaustive inventories of methane emissions and for developing the most effective responses. Monitoring provides a foundation for locating sources, identifying big emitters, designing the most effective policies, and then ensuring they are working. A global independent multi-tiered monitoring system would increase global pressure on nations and encourage the implementation of prudent policy decisions.^2,41−43^

## 2 Estimating Methane Emissions Using Top-Down and Bottom-Up Methods

### 2.1 Complementary Top-Down and Bottom-Up Approaches

Top-down and bottom-up methods of measuring anthropogenic GHGs can complement each other and reduce uncertainty in estimating methane emissions. Top-down methods have been significantly improved over the past decade, and there is increasing confidence in these methods as comparisons between and among satellite and airborne methods show general agreement.^20,35,44−49^ This recent development and ongoing refinement of top-down methods make them an increasingly popular tool for independent confirmation of anthropogenic carbon dioxide and methane emissions. On the other hand, bottom-up methods, while useful for identifying mechanisms and processes leading to emissions, often suffer from a number of potential sampling biases resulting from nonrepresentative sampling in time and space.

**Figure 1:**
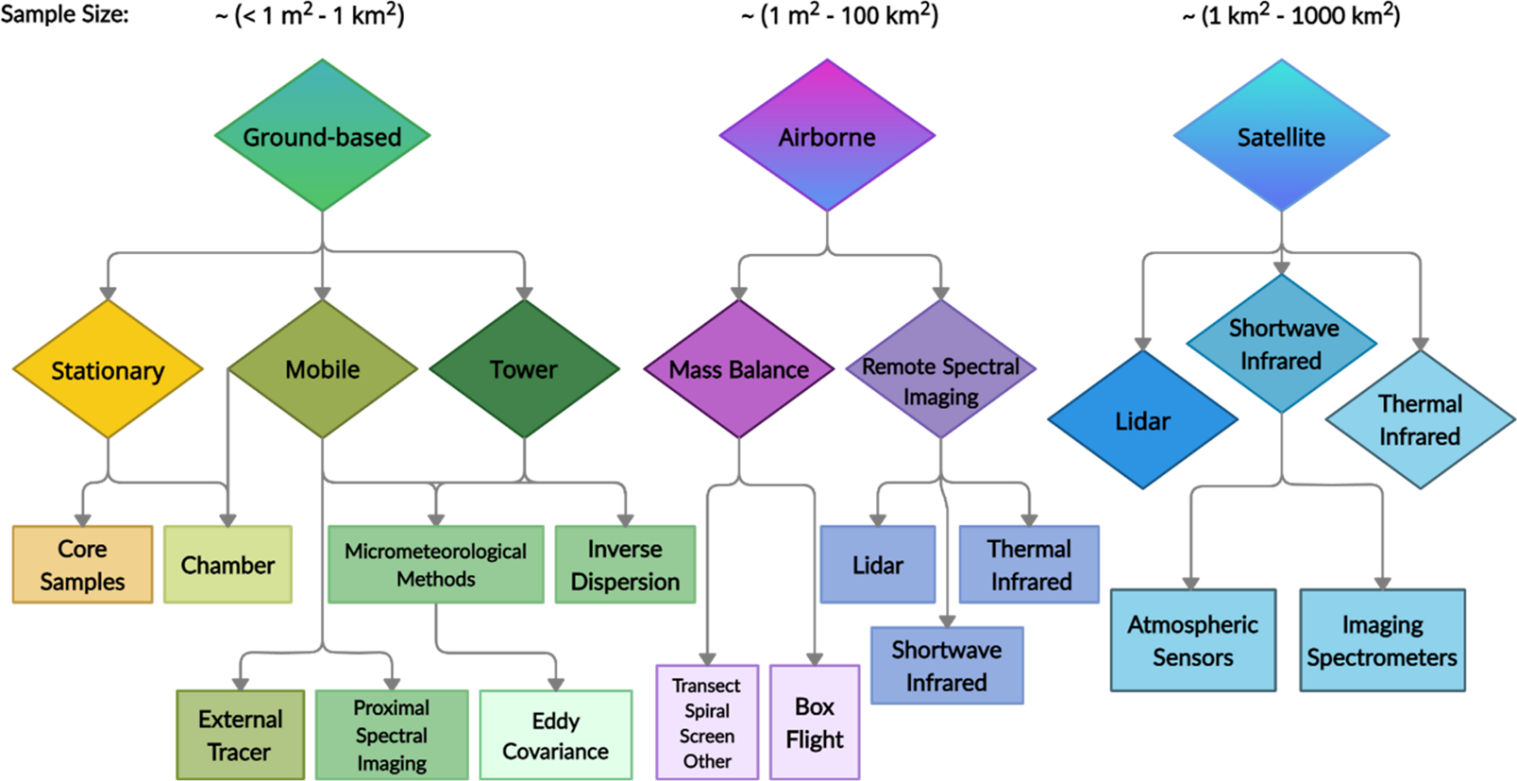
Taxonomy of sampling platforms and methods for estimating atmospheric methane emissions, arranged left to right according to approximate spatial sampling scale. Approximate ranges in the size of each method’s sample (e.g., pixel size or smallest resolvable sampling area) are provided at the top of each branch. Methods vary from bottom-up (toward the left) to top-down (toward the right). Note that some sensors and platforms (e.g., many airborne sensors) can be used for both extrapolation and downscaling and so can be considered as top-down or bottom-up, depending upon the analysis used. Tall towers are an exception to the ground-based sample size range as they often utilize inverse dispersion modeling and cover areas approaching the upper spatial scale of satellite methods. More color saturated upper branches depict the main method subcategories, while the lightly colored lower branches provide examples of techniques and data types.

Top-down and bottom-up approaches to measure gas fluxes can be addressed using combinations of ground-based, airborne, or satellite approaches, which are examined in this review. While satellites are generally used for top-down analyses, and ground-based sensors for bottom-up analyses, airborne sensors and platforms can potentially be used for both, providing a unique bridge between scales and sampling approaches. A taxonomy (Figure 1) illustrates methods for quantifying atmospheric methane emissions according to approximate spatial sampling scale, which tends to correlate to temporal sampling scale.^25^ These include direct, proximal, and remote methods to quantify methane concentration and flux. Most of these methods are based on spectroscopic determination of methane absorption features, some using imaging and some based on nonimaging methods. Spectral imaging methods are unique in their ability to capture unknown, or anomalous point sources at the proximal, remote, or orbital level. Table 1 discusses the main methods from Figure 1 and summarizes their key strengths and weaknesses. The purpose here is to provide an overview of the current state of methane monitoring technologies over a range of scales that, when combined, can provide a more complete picture of the methane budget. For a more detailed quantitative analysis of satellite capabilities and their uses for integrating top-down and bottom-up emissions budgets, see Jacob et al.^50^

**Table 1:** Descriptions, Strengths, and Limitations of the Main Methods for Ground-Based, Airborne-, and Satellite-Based Methane Emission Estimation Illustrated in the Taxonomy (Figure 1)

method	discussion	strengths	limitations	main reference(s)
Ground: Core Samples	Measured methane concentrations from core samples are used to approximate the release budget at a facility and extrapolate overall emissions released by the extent of oil processing.	Provides a unique approach to estimating emissions from below the ground. Used to estimate the regional methane emission budget constraints from rate of extraction and processing.	Limited by the correlation between concentrations measured in samples and emissions released during the processes. Requires multiple samples from sites.	(Johnson, Crosland et al. 2016)
Ground: Chamber Based	Directly measures emission concentration and flux rates from a local source, which can be extrapolated to larger areas.	High certainty of emission estimation within the sample. Method is independent of atmospheric modeling methods. Can sample at night.	Limited to small scales of sample and is very reliant on well-developed sampling schemes. Experiences bias from chamber artifacts. Criticized for missing sources and variability in regional estimates. Difficulty capturing sporadic emissions.	(Jeong et al. 2019; Chaichana et al. 2018)
Ground: Proximal Spectral Imaging	Proximal ground-based imaging spectroscopy to quantify and characterize plumes. Reliant on isolating the absorption lines from background noise.	High spatial and spectral resolution to identify samples at near ambient levels. Does not rely on external sources for wind measurements. More cost-effective than most airborne sampling.	Uncertainties increase as temperature contrast of GHG to the background decreases. Cannot quantify large areas and is restricted by the distance of sampling.	(Gålfalk et al. 2017)
Ground: External Tracer	Models emission concentrations from the downwind distribution of known concentrations of a tracer gas from a plume source.	Well developed, simplified method. Can handle complex sources and measure total emissions from a source area.	Labor intensive and dependent on access to facilities. Requires consistent, stationary wind and stable atmospheric conditions. Method breaks down if the desired gas does not follow the same dispersion as the tracer gas.	(Roscioli et al. 2018; National Academies of Sciences, Engineering 2018)
Ground: Inverse Dispersion	Directly measures concentrations of downwind plumes to model the upwind emission rate.	More cost-effective sampling than airborne methods and more robust than tracer or chamber-based methods. Can be used to quantify temporal trends.	Larger errors can occur from nonstationary wind and plume sources. Difficult to quantify and isolate complex, mixed sources. Reliant on externally modeled atmospheric conditions that may not match sampling conditions.	(Flesch et al. 2005; National Academies of Sciences, Engineering 2018)
Ground: Micrometeorological measurements (e.g., eddy covariance)	Models flux from direct measurements of gas concentration and high frequency wind (eddy) speed and direction in the appropriately scaled area of sampling.	Largest ground-based scale of atmospheric sampling. Ideal for capturing temporal trends. Measures uptake as well as loss. More cost-effective method of sampling large areas than airborne sampling.	Constrained by the need for homogeneous terrain and stable atmospheric conditions for low error in estimates. Requires an extremely rapid-response sampling device, which is expensive.	(Chaichana et al. 2018; Davidson et al. 2002; National Academies of Sciences, Engineering 2018)
				
Airborne: Mass Balance	Airborne measurements for quantification of localized, or regional plumes.	Can attain very low uncertainty (∼2%) in estimating fully captured stationary plume sources. Provides ideal in-depth modeling of regional emissions. Used for validation of both ground-based and satellite methods.	Costly sampling that requires known sources. Limited by boundary layer height. Issues with external sources, shifting plumes, and widely dispersed ground sources. Dependent on good extrapolation from lowest flight path to the ground.	(Conley et al. 2017; Gordon et al. 2015; Erland et al. 2022)
Airborne: Remote Spectral Lidar	Wavelength modulation spectroscopy measurements from a gas-absorbing laser scanner.	Can be used to quickly characterize and capture unknown emissions from a region. Under ideal conditions can obtain <10% uncertainty.	Aircraft vibration can create stripes in the data. Dependent on accurate geolocation and wind speed data.	(Bartholomew et al., 2017; Johnson et al., 2021)
Airborne: Remote Spectral Imaging	Absorption imaging spectroscopy of reflected solar radiance or thermal emissions to capture regional or facility emissions.	Quick sampling. Best for large mapping of plumes in a region. Can identify unknown sources such as leaks. Avoids temporal issues inherent to mass-balance methods.	Costly sampling, limited to appropriate meteorological conditions Requires multiple sampling to determine persistence of a source.	(Frankenberg et al. 2016; Bartholomew et al. 2017; Erland et al. 2022)
				
Satellite: Remote Spectral Imaging^a^	Uses imaging spectroscopy to quantify absorption features in reflected solar radiance to capture global, regional, and local estimates of emissions.	Repeatedly samples global and regional emissions. Provides independent monitoring without the need for site permission.	Currently has coarse spatial resolution. Some methods are restricted by spectral interference and difficult sampling of dark scenes, or high reflectance scenes such as snow, or water.	(Cusworth et al. 2019; Kort et al. 2014; Jacob et al. 2016)

a See Table 2 for a further breakdown of the satellite methods.

### 2.2 Ground-Based Methods

**Figure 2:**
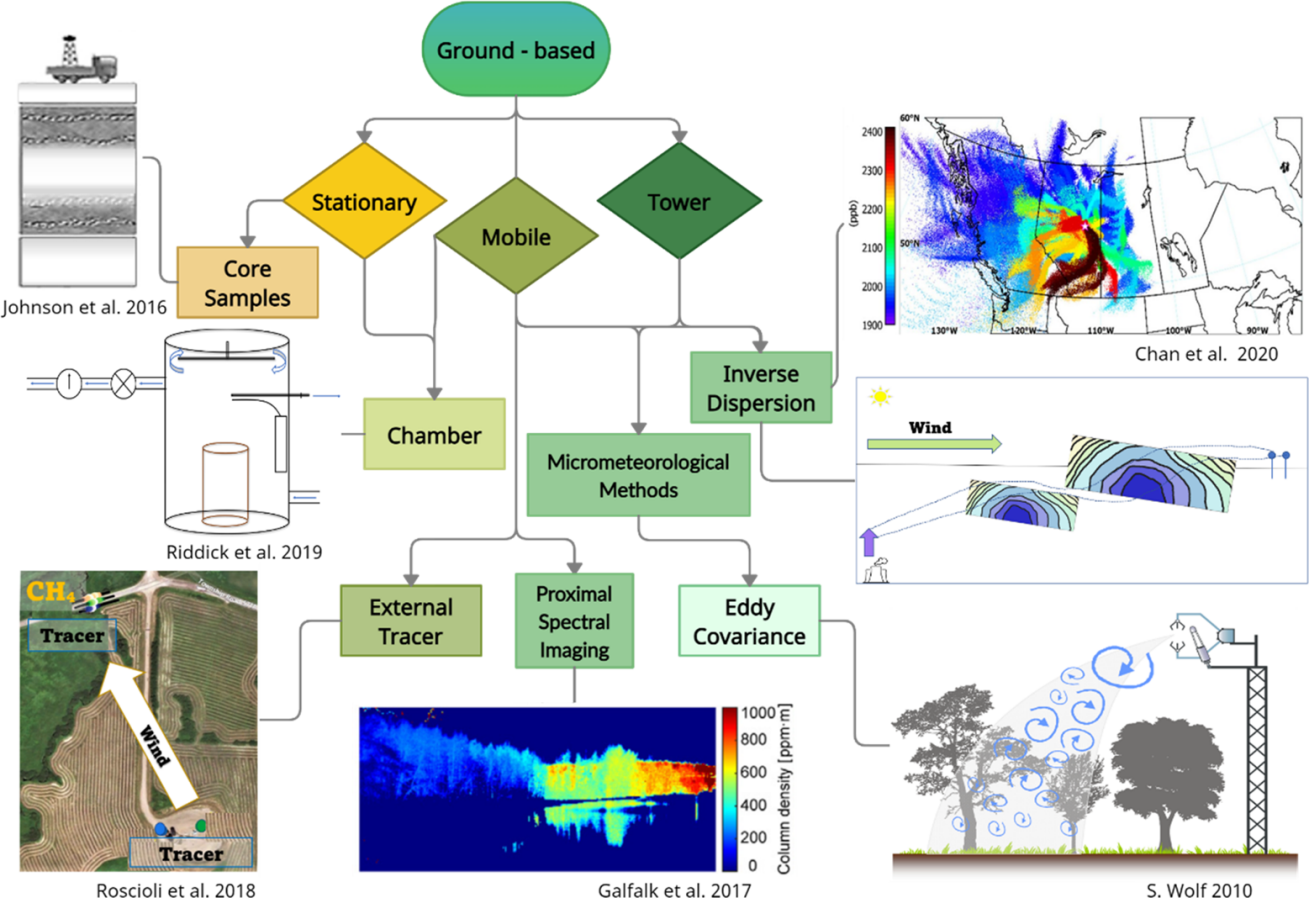
Taxonomy of main ground-based methods for measuring methane emissions. Figures adapted* from ref s^33,38,39,52,69,127^. *Ref (69) reprinted with permission from Elsevier.

Ground-based methods are currently the most direct route for continuous estimation of point source and regional anthropogenic emissions. Figure 2 illustrates the types of methods that can be applied through sampling on stationary towers, fixed sensors, portable hand-held devices, or automobile laboratories.^51^ Tall towers provide continuous observations for estimating regional emissions, and networks of tall towers can provide ground-based, top-down atmospheric estimates.^33^ Mobile laboratories are often used to provide numerous spatially explicit eddy covariance, flux chamber, or tracer gas samples to scale up to site and regional estimates.^29,52^ Site features, such as tank vents, well pads, pump stations, or shutoff valves, are often best estimated by ground-based measurements for pin-pointing source emissions due to their small spatial scale.^52^

Ground-based, bottom-up methods can be more challenging than airborne methods to quantify emissions from a regional area as they are dependent on good extrapolation and modeling.^52,53^ Ground-based measurements also provide critical quantification of local emissions and validation data for top-down methods; tower data are essential for validation, and surface measurements provide validation for calculations used in mass-balance airborne methods.^23,43,54,55^

In addition to the emission estimation methods shown in Figure 2, air samples of methane can be used to attribute sources by analyzing the isotopic signature posthoc using a laboratory isotope-ratio spectrometer.^56^ Anthropogenic methane emissions from pyrogenic and thermogenic sources are enriched in the δ^13^C isotopic signature compared to biogenic sources such as wetlands.^56^ This enables areas that may have mixed sources, to determine the ratio of biogenic compared to anthropogenic emissions and better attribute the source of the methane.^32^ Furthermore, there are many other direct measurement methods used for mapping concentrations, detecting leaks, and evaluating health risks of emissions such as hi-flow sampling^57−59^ and the OTM-33A as standardized by the Environmental Protection Agency.^60,61^ While these methods can be easy to use and provide rapid first assessment of the source,^60^ they often require access to industry facilities, are best suited for estimating small point sources with minimal spatial and temporal variability, and tend to have large uncertainties.^62^

#### 2.2.1 Core Samples

A novel approach to quantifying point source, fugitive methane emissions from heavy oil mine sites has been introduced by Johnson et al. 2016. Fugitive emissions can be estimated by quantifying the amount of methane present *in situ* and subtracting the remaining gas after processing.^39^ Measurements are limited by the reliance on industry to obtain the core samples and currently have high rates of uncertainty for estimates (34–69%).^39^ This method presents a unique way of constraining a budget for atmospheric methane emissions from below the ground, rather than from on or above the ground, and estimates the potential emissions of proposed mining sites.^39^

#### 2.2.2 Chamber Methods

Chamber measurements are a simple way of measuring fluxes with few modeling assumptions and as such are commonly used for estimates by industry.^25^ Flux chambers are ideally suited to measuring small, contained sources.^38,63^ Chamber sampling methods provide a direct measurement of emissions from sources, and when instruments are mobile they can effectively sample a defined landscape;^64^ however, the spatial sampling range of an individual chamber is very small. To address this limitation, Jeong et al. 2019 assessed interpolation methods of point sampling to determine an efficient sampling scheme that maximizes distribution while minimizing the number of samples for a given spatial interpolation.^65^ Poor design of stratified sampling, and/or using a limited number of samples can result in large over- or underestimation of fluxes.^63,64^ Chamber artifacts must also be minimized when placing the chamber as the act of measuring can bias the flux concentrations by perturbing the sample area.^66^ Closed chamber methods can be successful in accurately measuring emissions from well-known temporally distributed emissions.^39^ Dynamic flux chambers have been successfully used in a campaign to estimate fugitive methane emissions from abandoned wells by fitting the chambers over the wells to estimate leakage.^38^ Issues arise in extrapolating samples from complex sites with large sporadic emissions, so chamber methods are not well suited for estimating annual emissions from an entire oil production site.^39,63^

#### 2.2.3 Proximal Spectral Imaging

Proximal imaging spectroscopy methods are becoming increasingly popular as a cost-effective way to capture unknown, fugitive emissions, produce imagery to characterize sources, as well as estimate methane emissions.^67−69^ A recent hyperspectral method can capture both known and unknown sources by simultaneously sampling meteorological variables, and methane and nitrous oxide in the 1.0–5.5 μm midwave range and the 7.7–9.5 μm longwave range to estimate emission flux from a single instrument.^69^ Hand-held and mobile spectral devices are also being developed that utilize differential absorption lidar (DIAL) to detect, locate, and quantify carbon dioxide and methane emissions from oil and gas facilities.^35,68^ Both methods are used to scan scenes, take “snapshots” to locate fugitive leaks, and assess source emissions. Ideal samples are captured when conditions allow for a distinct emission enhancement that can be easily separated from the background.^69^ Methods by Galfalk, Olofsson, and Bastviken 2017 produce near-ground measurements of the distribution of GHGs from various environments and sources and processes images compiled as movies to characterize plume behavior.

#### 2.2.4 External Tracer and Dispersion Models

The tracer-gas ratio method has been used to measure emissions from fossil fuel extraction sites for over 20 years.^52^ It applies the working assumption that a known gas has a dispersion distribution identical to the desired emission gas and estimates emissions based on a known ratio of concentrations.^52^ Dispersion models can be applied to, or combined with tracer measurements, eddy covariance, or spectral laser measurements to extrapolate given samples to the distribution of emissions from a site.^53,65,70^ Inverse dispersion modeling works by backward sampling a known concentration to estimate emission information from an upwind source.^53^ A recent study aggregated 6650 mobile ground-based measurements using an external tracer and inverse dispersion modeling to produce estimates from the upstream Canadian oil and gas and found that inventories underestimated methane emissions by a factor of 1.5.^36^ Methods for up-scaling to larger regional methods are still being developed, and methods are best used for well-known, consistent concentrations.^25^ Both external tracer and dispersion modeling benefit from averaging; therefore, the greater the spatial distribution and number of samples, the lower the error.^30,33,52^

#### 2.2.5 Tower Sampling and Micrometeorological Methods

Flux towers can directly measure methane and carbon dioxide fluxes as they occur using eddy covariance techniques. They are able to detect small changes in net ecosystem exchange, or the flux between the atmosphere and vegetation, by measuring eddies to calculate the covariance between GHG mixing ratios and vertical wind velocities.^71,127^ Measurements can be analyzed independently or combined with samples acquired using other ground-based methods to model flux within the area of sampling. Flux towers are often constrained by the scale of sample, a lack of homogeneous terrain, and a stable atmosphere. For example, rugged mountainous areas with volatile atmospheric boundary layers make for inopportune flux tower locations. In spite of these limitations, flux towers are emerging as a fundamental source of GHG emission data as this technology becomes more available.^72^ There is an increasing global abundance of flux tower sites providing continuous large scale measurements, and international networks such as FLUXNET are enhancing the value of using the eddy covariance method for validating airborne and satellite data.^71,72^ In addition to sampling local areas with eddy covariance, tall towers can be used to sample regions comparable in size to some of the larger satellite sampling scales. Regional Bayesian inverse dispersion modeling was recently successful in modeling eight years of observations from four tall tower sites to estimate annual methane emissions from Alberta and Saskatchewan, and it found anthropogenic emissions from the oil and gas sector to be almost twice those reported in Canada’s National Inventory.^33^

### 2.3 Airborne Methods

**Figure 3:**
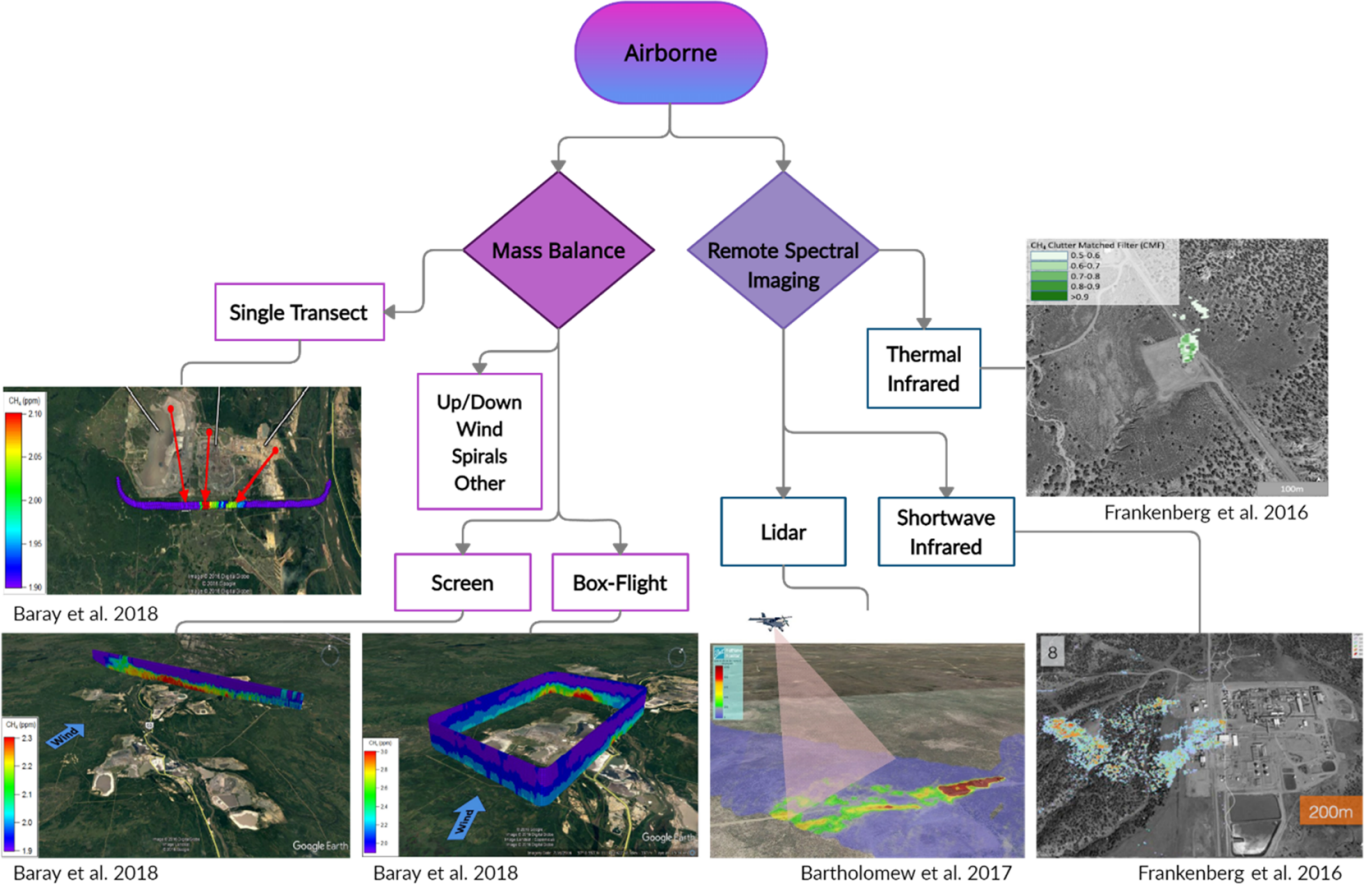
Taxonomy of main airborne methods for measuring methane emissions. Figures adapted* from refs (20,28,84). *Ref (84) reprinted with permission from SPIE.

Airborne measurements are used to characterize and validate site, area, and regional methane emissions.^29^ They provide an intermediary sampling scale that can attain detailed quantification of emission budgets from sites and sample large regional areas.^24,49^ Methods for sampling atmospheric GHGs from the air tend to fall into two major categories: (i) remote spectral imaging methods and (ii) mass-balance spectroscopic methods. Figure 3 illustrates the difference between the sampling schemes and imagery produced from the two approaches. Accurate wind measurements are paramount to all airborne methods. Airborne methods are limited by airspace restrictions and can be costly and time intensive, which makes them ineffective as continuous monitoring methods, but they provide in depth emissions estimates for supplementing and auditing emissions inventories, as well as follow up to emission hotspots flagged by satellite data.^73^ Because of the high cost of repeat visits, airborne methods are not always able to assess the temporal variability of emissions and rely on ground-based or satellite measurements to upscale hourly emission rates to monthly and yearly estimates for inventories.^31,49,74^

Other methods are being developed in addition to those outlined in Figure 3, which may provide additional measurement methods as utility improves. Drones have become popular in some fields to sample methane emissions hotspots from the air, and progress is being made in emissions estimation; however, they currently sample at too small a scale to get meaningful data for monitoring oil and gas facilities.^75^ As they avoid some ground access and safety issues, and to the extent that they can sample large areas over facilities, drones and airborne methods are often ideal tools for monitoring pipelines for leaks.^20,75^ Airborne eddy-covariance methods can also provide unique surface-atmosphere exchange mapping of an area.^76^ While this method has been used as an alternative and complementary airborne method for characterizing emission dynamics over oil and gas regions,^77^ it is more suited for characterizing spatially extensive ecosystems with diffuse emissions,^78^ and as such can be difficult to use when trying to quantify the large sporadic point source methane emissions which are common to the oil and gas industry.

As with satellite methods, spectral airborne methods often complement ground measurements, particularly when coordinated with ground measurements, such as those made with the global flux tower network (FLUXNET),^71,79^ a global, multiecosystem network of ecosystem-atmosphere gas fluxes, which has begun to consider methane fluxes in addition to carbon dioxide.^80^ 2019 was labeled the “Year of Methane” by AmeriFlux, the American branch of FLUXNET, to emphasize the emerging importance of these measurements.^72^ Such ground networks provide an example of how direct flux assessment from the ground with remote sensing methods creates a powerful synergy that can assist with validation and upscaling, and could be extended to include anthropogenic emissions.

#### 2.3.1 Mass-Balance Methods

Mass-balance methods most commonly capture point and area source plumes by flying in transects, screens, or box-flights that constrain the emission plume as it traverses the flight path around the source.^23,24,81,82^ Airborne mass-balance flights can sample following several sampling schemes; the simplest are single transects and screens which are faster, but have large errors (25–60%), or there are more complex time-intensive box-flights which surround an emissions source and attain a lower estimate error (∼2%).^23^ Two box-flight mass-balance algorithms, the Top-down Emission Rate Retrieval Algorithm (TERRA) and SciAv (Scientific Aviation) model, have recently been applied to box-flight patterns when an aircraft encircles a source to estimate emissions by calculating the flux through the boxed-in source.^20,23,29,31,46,49,82,83^ Full capture at the top of the box is often attained by operators flying laps up to the top of the (stable) atmospheric boundary layer, which typically caps the top of an emission plume.^23^ Due to minimum flight height restrictions, a gap between the surface and the flight box is inevitable. Extrapolation to the ground has been shown to often be the largest error source, nearing ∼30% in both models when the bottom of the plume is not captured.^23,82^ Currently, mass-balance box flight methods can attain a lower uncertainty in emission estimates than the remote spectral imaging due to smaller background and wind measurement uncertainties, but require prior understanding of plume sources and stationary conditions, take longer, and are often more costly.^25,74^

#### 2.3.2 Remote Spectral Imaging

Remote spectral imaging from the air can be used to quickly cover broad areas providing snapshots of plumes to estimate emissions, visualize the large sporadic leaks that are common to fossil fuel extraction sites, and depict a distribution of plumes to characterize an area of interest.^20,84−86^ This method samples within seconds and therefore avoids the temporal limitations inherent to airborne mass-balance methods, and its ability to quickly capture unknown leaks and emission sources provides a cost-effective method for reducing GHG emissions over large regions.^74,85,87^ By rapidly addressing methane leaks, a recent study of the Southern Midland Basin using an airborne hyperspectral imaging instrument recovered costs of their first flight campaign within 5 days.^88^ However, remote spectral sampling is still collected on a “campaign” basis and is not typically used for routine emissions monitoring.^46^ As remote spectral imaging methods advance, and error is reduced, these methods herald the possibility for quick, exhaustive regional sampling to identify both known and unknown sources.

Passive airborne methods utilizing shortwave or thermal infrared spectroscopy have been developed to map carbon dioxide and methane concentrations, and detect and estimate emission rates over large areas.^20,85,89,90^ A shortwave infrared instrument, the Airborne Visible InfraRed Imaging Spectrometer - Next Generation (AVIRIS-NG^91^) (NASA Jet Propulsion Laboratory, Pasadena, CA, USA), can attribute and estimate point source emission rates as small as 1–3 m with a detection limit of 2 kg h^–1^ to 5 kg h^–1^ depending on wind speed with uncertainties of ∼30%.^46,74^ The Kairos LeakSurveyor (Kairos Aerospace, Mountain View, CA, USA) is a smaller, relatively inexpensive instrument designed for commercial deployment, rather than research purposes of the AVIRIS-NG.^87^ It calculates a methane emission rate, adjusted to avoid the need for wind speed measurements, with a minimum detection level of 5 kg per hour per meter per second of wind with a method error of ∼30–40% uncertainty.^85^ Shortwave infrared airborne sampling can be confounded by spectral inference from the surface,^92^ or reflectance from features such as tailings ponds.^93^ Thermal infrared airborne sampling using instruments such as the Hyperspectral Thermal Emission Spectrometer (HyTES) (NASA Jet Propulsion Laboratory, Pasadena, CA, USA), provide a useful complement to shortwave infrared as they can sample when spectral interference impedes infrared sampling, but have coarser spatial resolution, and gas retrievals have significant uncertainties associated with air temperature, surface emissivity, and atmospheric water vapor, which makes them less ideal for emission estimation.^20^ Several airborne imaging campaigns in California, Colorado, Texas, and New Mexico have been successful at mapping large regional methane plumes and estimating point sources and are increasingly becoming a popular method.^35,43,74,85,86,94^

Airborne Lidar methods are active methods that are ideal for quickly sampling large areas for unknown sources and to characterize regional patterns in background and anthropogenic emissions.^84,95,96^ The IPDA Lidar (Ball Aerospace & Technologies Corp, Boulder, CO, USA) provided one of the first opportunities to the measure regional and temporal variability in background concentrations, which had largely been restricted to ground-based methods and modeling.^84^ Aircraft vibrations creating stripes in the Lidar data, determining the ideal sampling beam diameter, and dependence on accurate geolocation data are weaknesses of the method.^84^ The Gas Mapping Lidar (Bridger Photonics, Bozeman, MT, USA) has a spatial resolution of 2 m and can estimate methane emissions with an uncertainty range of 31–68%.^35^ In 2019, the Bridger Photonics lidar completed a campaign across 167 geographically distinct sites in British Columbia and estimated 80 methane sources with a range in emission averages from 0.5 to 399 kg h^–1^.^97^ While estimate uncertainties are on average higher for airborne lidar than shortwave infrared, lidar methods can currently attain a lower detection limit of emission plumes (∼0.6 kg h^–1^ versus 2 kg h^–1^), but typically fly at lower altitude and have a smaller image swath, resulting in less ground coverage.^35,74^

### 2.4 Satellite Methods

**Figure 4:**
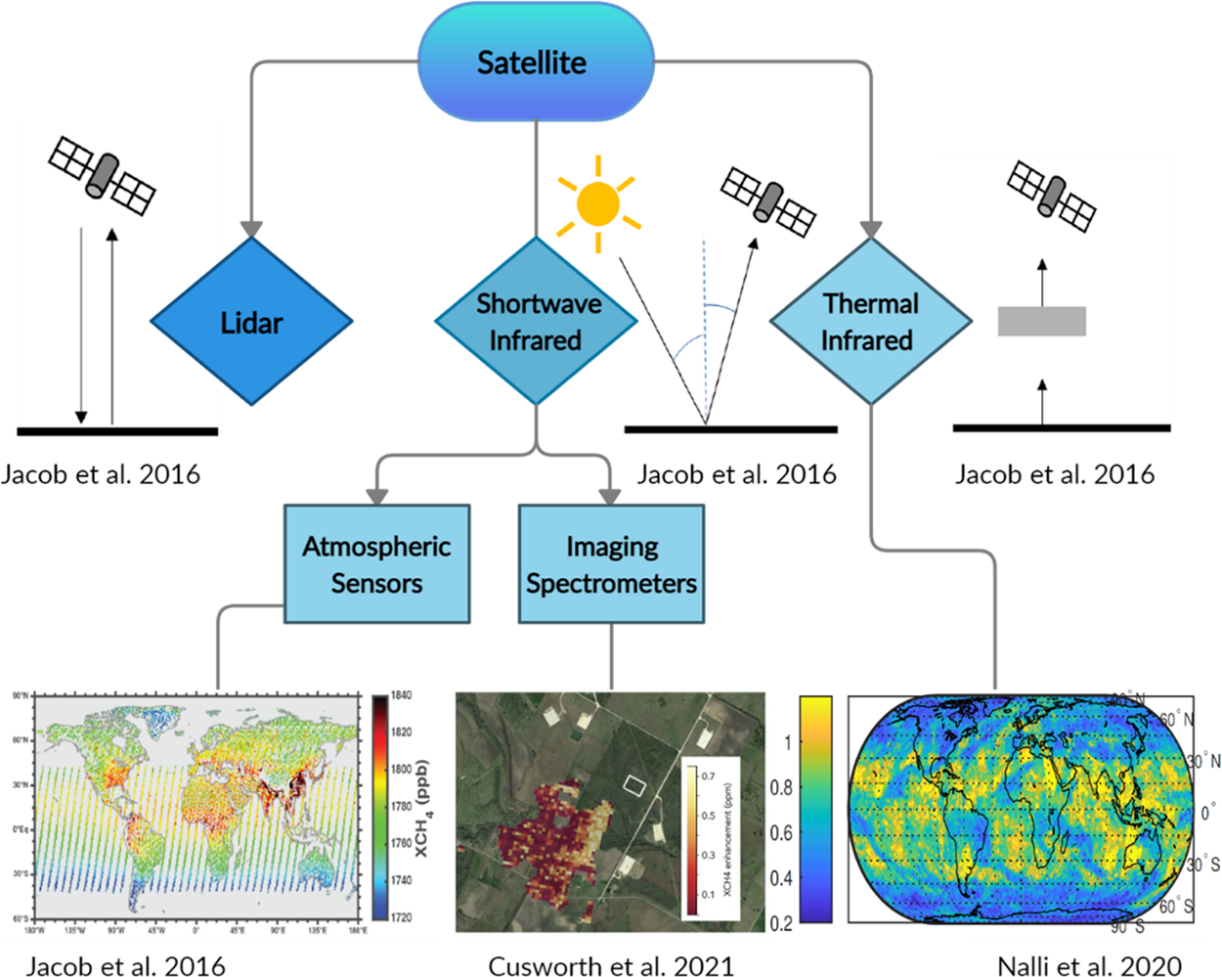
Taxonomy of main satellite methods for measuring atmospheric methane emissions. Figures adapted from refs (44,55,86).

Satellite measurements allow for repeated sampling over regional to global scales and can produce estimates where it is not feasible to sample using other methods.^25^Figure 4 depicts current methods for satellite observations of atmospheric methane. Satellites are generally used for top-down methane measurements,^50^ and the next decade of planned satellite imaging spectrometers (ex. EnMap, PRISMA, Carbon Mapper) will continue to close the uncertainty gap between the global top-down and bottom-up methane budget.^98^

Each GHG has its characteristic absorption bands, and each satellite instrument measures absorption features in slightly differing spectral ranges often called fitting windows. In general, methane is measured in the shortwave infrared range (SWIR) around 1.65 or 2.3 μm wavelengths, or in thermal infrared (TIR) wavelengths around 8 μm.^44^ Most atmospheric sensing satellites passively measure the absorption of radiation by solar backscatter using large wavelength ranges and at a high spectral resolution. Satellite imaging spectrometers aim to address the desire for fine pixel resolution to resolve point sources, while increasing the spectral resolution.^94^ Lidar provides coarser active measurements of emissions using its own laser light source, freeing it from sun illumination, and can therefore measure during the day or night.^99^ This means satellite Lidar methods using integrated path differential absorption will uniquely provide the ability to measure methane emissions nearer the poles when daylight is limited.^44^ TIR methods are not restricted by spectral interference and can sample dark sources, but tend to have poor sensitivity of lower tropospheric emissions which can make point source estimation difficult as they often miss plumes at the surface.^44^Table 2 summarizes examples of the instruments used in the methods illustrated in Figure 4 for measuring carbon dioxide and/or methane emissions from space. For a more exhaustive and quantitative assessment of all current and future satellite methods, see Jacob et al.^50^

**Table 2:** Examples of Instruments for Measuring Methane (CH_4_) and Carbon Dioxide (CO_2_) Using the Satellite Methods Depicted in Figure 4^a^

method	instrument	gas	discussion	resolution^b^ spectral (nm^c^)^d^ spatial (km^2^)	main reference
SWIR: Atmospheric Sensor	SCIAMACHY	CH_4_, CO_2_	Large spatial coverage and achieved first global mapping (2002–2012)	0.4, 1.4, 0.2	(Buchwitz, et al. 2005)
				30 × 60	
	GOSAT	CH_4_, CO_2_	Fine spectral resolution, measures select global locations (2009–present)	0.02, 0.06, 0.10	(Kataoka, et al. 2017)
				10 × 10	
	GHGSat	CH_4_, CO_2_	Constructed with a very fine pixel resolution to measure facility scale emissions. Claire (2016–present) Iris (2020–present)	0.1	(Jervis, et al. 2021)
				0.05 × 0.05	
	TROPOMI	CH_4_	Can produce daily estimates of surface emissions of methane. (2017–present)	0.25	(Hu, et al. 2018)
				7 × 7	
SWIR: Imaging Spectrometer	PRISMA	CH_4_, CO_2_	The first two main satellites launched using hyperspectral sampling. Attains fine pixel resolution. (2019–present)	10	(Cusworth, et al. 2019)
	EnMap			0.03 × 0.03	
	EMIT	CH_4_, CO_2_	Will attain moderate pixel and spatial resolution. Data will be publicly available.	7.4	(Ayasse, et al. 2019)
				0.06 × 0.06	
	Carbon Mapper	CH_4_, CO_2_	Will attain fine pixel and spatial resolution. Data will be publicly available. (Not launched yet.)	6	(Carbon Mapper)
				0.03 × 0.03	
TIR	MethaneSAT	CH_4_, CO_2_	Intended for broad monitoring with point source estimation. Data will be publicly available. (Not launched yet.)	0.3	(Rohrschneider, et al. 2021)
				0.01 × 0.04	
	TES	CH_4_	Achieved the smallest TIR pixel size with fine precision (2004–2011)	0.8	(Worden, et al. 2012)
				5 × 8	
	IMG	CH_4_, CO_2_	Provided the first satellite sensing of methane (1996–1997)	0.1 cm^–1^	(Shimoda, Ogawa et al. 2000)
				8 × 8	
	CrIS	CH_4_, CO_2_	Hyperspectral infrared sounding method with the best vertical resolution through the troposphere. (2011–present)	0.625 cm^–1^	(Nalli, et al. 2020)
				14 × 14	
Lidar	Merlin	CH_4_	Intended to measure in dark conditions at a fine resolution. (Not launched yet.)	N/A	(Wührer, et al. 2019)
				200 μm	

a Full names of abbreviations: SCanning Imaging Absorption spectroMeter for Atmospheric CartograpHY (SCIAMACHY), Greenhouse gases Observing SATellite (GOSAT), Greenhouse Gas Satellite (GHGSat), TROPOspheric Monitoring Instrument (TROPOMI), PRecursore IperSpettrale della Missione Applicativa (PRISMA), The Environmental Mapping and Analysis Program (EnMAP), Technology Experiment Satellite (TES), Interferometric Monitor for Greenhouse gases (IMG), Cross-track Infrared Sounder (CrIS), Methane Remote Sensing Lidar Mission (Merlin).

b When the instrument measures both CO_2_ and CH_4_ at varying spectral resolutions, resolution is given as band1, band2, and band3.

c Except where otherwise noted.

d Given as the full width at half maximum (FWHM).

As methods improve, the trade-offs between spectral and pixel resolution, and global sampling versus point source attribution are becoming more relaxed.^42^ Over the past decade, several measurements have been made by satellites to estimate point-source methane emissions with increasing pixel and spectral resolution.^21,50,73,98,102,109−111^ In the fall of 2020 using a controlled release, GHGSat (Iris) accurately measured the smallest methane emission plume from space which was also validated by a simultaneous aircraft measurement (260 kg h^–1^).^112^ Hyperspectral (high spectral resolution) and ultraspectral (extremely high spectral resolution) sampling will increase the accuracy and improve the precision of point source estimation by increasing the independent measurements within the given spectral window and allowing for better resolution of absorption features.^55,94^ A study released in early 2022 used multiple hyperspectral satellite instruments (e.g., PRISMA) in combination with TROPOMI data to capture unknown leaks causing super-emissions from pipelines in one of the world’s largest methane producing regions.^21^ Open access satellite technology with increased pixel resolution and larger fields of view, such as Carbon Mapper and MethaneSAT, will provide the remarkable capability to produce independent point source GHG measurements from space of international sites that may lack transparency of emissions.^105,106,110^ At the time of writing, geostationary methods such as the GeoCarb mission have not been launched but if successfully implemented will allow for continual monitoring of a chosen region, rather having a predictable overpass time for each orbit.^113^ Continuous monitoring and time-averaged satellite emissions estimates will improve the estimation of annual methane emissions from facilities for monitoring and reporting, and validation.^111^ The projected refinement of satellites in the next decade will enable temporally and spatially continuous point source emission quantification and attribution. As these satellite instruments improve, we can envision satellite sensors being used not just for top-down, but increasingly for bottom-up, emissions assessment, at least for large emission sources.

## 3 Transparent Monitoring for Rapid Methane Reductions

As demonstrated in this review, technology for estimating methane emissions has improved dramatically in the past decade, and a wide array of methods is now poised to provide a solid and verifiable foundation to enable rapid abatement of significant components of the anthropogenic methane budget. We have the tools to measure across spatial scales using the same fundamental spectral imaging technology, providing a level of methodological consistency across scales that has been previously lacking. The monitoring capabilities of multi-tiered spectral imaging, supported by other methods for more in-depth and precise analysis of known sources, has drastically increased our ability to detect, accurately estimate, and pinpoint abatable super-emissions.^20,21,42,43,46,50^

The need for substantial reductions of methane emissions to combat global warming quickly and efficiently was placed on the global stage in 2021. In the fall of that year, at the COP26 summit in Glasgow, 103 nations signed the Global Methane Pledge committing their governments to a collective goal of reducing methane emissions by a minimum of 30% of 2020 levels by 2030.^114^ This reduction goal will require immediate and coordinated action. In the past, uncertainty in the methane budget may have provided an argument for inaction, but that will no longer be the case, given the emergence of new sampling methods discussed here, along with the clear demonstration that large emitters can now be readily identified and targeted for abatement. While the science is clear, and appropriate sampling technology is now available, it remains to be seen if nations will follow through on their pledges.

A brief consideration of the Canadian Oil Sands example can be instructive and sobering. During the COP26 summit, Canada committed to an emissions cap on the domestic oil and gas sector, with the goal of achieving net zero by 2050.^115^ Yet, in the spring of 2021 Canada’s official opposition party voted to not recognize the climate crisis as real, a position at odds with both recent policy decisions and the established scientific certainty.^116^  The Government of Canada and the Province of Alberta have adopted different and potentially conflicting emissions reduction regulations and targets, with increasing public disagreement between ministers, and while federal policies are found to be stronger, neither government is currently poised to achieve the 2025 target.^,117,118^ The Government of Canada has pledged to end federal financing for foreign fossil fuel projects in 2022.^119^ In 2019, the Government of Alberta initiated a “fight-back” policy which included creating the Canadian Energy Centre to promote Alberta’s O&G sector, and a public inquiry into “anti-Alberta energy campaigns” and foreign investment in environmental initiatives.^120,121^ Oil Sands monitoring has often been discontinuous, leading to concerns about credibility and transparency.^122^ The creation of an independent environmental monitoring agency in Alberta was legislated in 2013, functionally established in 2015, then dissolved in 2016 and reincorporated into the Alberta Government as the Environmental Monitoring and Science Division (EMSD). Alberta's Oil Sands monitoring is currently under the Resource Stewardship Division. These recent developments suggest that environmental monitoring in the Oil Sands will continue to be a complex and contentious issue for the foreseeable future. Such national-level discord needs to be resolved to allow successful international action.^116^

Scientific autonomy from political mandates is essential to the credibility of monitoring and science programs. Instability, lack of scientific leadership, lack of clarity of purpose, and complex technical issues are a challenge for any long-term monitoring program,^122^ let alone one that appears to be at odds with a government’s stated public priorities and policies. Until recently, national and regional inventory estimates have largely been reliant on industry monitoring and reporting itself, which has clearly failed to attain clarity in GHG emissions budgets. A recent study found ground-based estimates differed in part due to a scarcity of publicly available data.^123^ The shift toward publicly accessible continuous satellite monitoring will enable more transparent and accurate emissions estimates. Furthermore, methane from the O&G sector tends to have only 20–35% emission persistence when resampling over an area,^74^ and therefore the ability of continuous satellite monitoring for large sporadic events, such as venting and leaks, with more in-depth airborne and ground-based follow up, will enable inventories to capture the more dynamic emissions that occur.^46^ Currently, nonprofits such as the Environmental Defense Fund are spearheading transparent monitoring, which is leading to increasing public pressure as scientific evidence of the importance of methane abatement builds.^106^

Given the advances in technology covered in this review, we are placed to make rapid progress if the national polices to make that possible are appropriately designed and implemented. An adaptive management approach that updates policy according to the latest accurate and transparent scientific findings may be needed, as has been accomplished with the Montreal Protocol and subsequent agreements to limit CFC reductions, leading to a gradual recovery of the global stratospheric ozone layer.^124−126^ Ultimately, the success of any framework will depend on the rigor of the science underpinning it, the use of appropriate technology enabling it, and adoption of a clear purpose and mandate. The technologies outlined here will play an essential role in attaining our common goal of lasting and meaningful reductions in GHG levels.

## 4 Emerging Technology: Closing in on the Top-Down and Bottom-Up Gap

Ground-based, airborne, and satellite methods of measuring anthropogenic methane emissions can be used to complement each other to fill measurement gaps, clarify assumptions, and reduce uncertainty in overall regional inventories. Advances in obtaining regular coverage using spectral airborne and satellite sampling methods will make these methods ideal for quantifying process emissions and sporadic flaring events to monitor changes and capture leaks, targeting the largest emitters, and enabling more accurate and complete estimates of methane fluxes. Given the wealth of data these methods provide, it is critical that stakeholders have a clear application for the data they collect, select technologies appropriate to the spatiotemporal distribution of the targeted emissions, and include independent data to validate estimates. Increased transparency and availability of industry data, such as site-level time-stamped operational data, would provide essential information to investigate discrepancies between methods and facilitate the most accurate emissions inventories.

International climate agreements have a history of poor implementation as nations sign and then back out as governmental priorities change.^22^ Such backtracking may become more difficult in an emerging world of accurate and transparent monitoring technologies, combined with a growing public demand for effective climate action. Significantly reducing methane emissions is an efficient and effective route to immediately combat climate change. The reduced uncertainty in top-down methods, increased confidence as more methods are found to agree, and refined detection thresholds mean we already have the tools to identify and combat methane super-emitters and make significant contributions toward reducing global warming. Emerging global frameworks utilizing multi-tiered continuous independent monitoring of anthropogenic methane emissions will provide vital transparently derived inventories to hold nations accountable to their international climate accord agreements.
